# Erdheim-Chester Disease Presenting as Bilateral Facial Masses: A case report and review of literature

**DOI:** 10.18295/squmj.6.2024.031

**Published:** 2024-08-29

**Authors:** Asim Qureshi, Abdulaziz Bakathir, Fizza Qureshi, Amanullah Beg, Asem Shalaby

**Affiliations:** 1 Department of Pathology, Oman Dental College, Muscat, Oman; 2 Department of Dental & Faciomaxillary Surgery, Oman Dental College, Muscat, Oman; 3 Department of Oral Surgery, Oman Dental College, Muscat, Oman; 4 Radiology, Sultan Qaboos University Hospital, Sultan Qaboos University, Muscat, Oman

**Keywords:** Erdheim-Chester Disease, Langerhans Cell Histiocytosis, Case Report, Oman

## Abstract

Erdheim-Chester disease (ECD) is a rare form of non-Langerhans cell histiocytosis with unknown aetiology. It is recently recognised to be neoplastic with genetic mutations affecting the mitogen-activating protein kinase pathway. We report a 49-year-old female patient who initially presented in 2012 to a tertiary care centre in Muscat, Oman, with bilateral facial masses. These were removed but later recurred over a period of 10 years. She then presented with xanthelasmas, bone lesions, secondary infertility due to hypothalamic hypogonadism, diabetes insipidus and Hashimoto’s hypothyroidism. The facial masses were biopsied and they showed classic morphological features in the form of diffuse infiltration by foamy histiocytes with scattered Touton type of giant cells, patchy lymphocytic infiltrates and dense fibrosis. The patient is stable and is being followed-up. The presented ECD case is particularly interesting due to the recurrent bilateral facial masses. To the best of the authors’ knowledge, this is the first documented case in Oman.

Erdheim-Chester disease (ECD) is a rare non-Langerhans cell histiocytic disease. It was first described in 1932 by William Chester while he was visiting Jakob Erdheim in Vienna, Austria. They reported the disease as “lipoid granulomatosis”.^1^ In 1972 Dr. Ronald Jaffe reported a third similar case and coined the name ECD.^2^ It is a rare disease that is being increasingly recognised; more than 1,000 cases have been reported in the literature over the last decade.^3^

This rare, potentially fatal multi-organ myeloid neoplasm occurs primarily in adults with a slight male predominance. It is believed to be under-diagnosed due to the wide variety of manifestations often mimicking other diseases and simultaneous involvement of multiple organ systems.^4^ The mean age of onset is 55–60 years. Clinical presentation varies depending on the extent and distribution of the disease and may range from asymptomatic incidentally diagnosed bone lesions to multisystemic, life-threatening forms with poor prognosis.

The diagnosis of ECD is based on a combination of histopathological, clinical and radiological features. It is reported that more than 95% of ECD patients have skeletal involvement. The commonest of which is bilateral and symmetric cortical osteosclerotic lesions of the diaphyseal and metaphyseal regions of the long bones especially the distal femur, proximal tibia and fibula. These radiological characteristics are highly suggestive of the disease.^5^ The extraskeletal manifestations may include exophthalmos, diabetes insipidus, interstitial lung disease, cardiovascular involvement, adrenal enlargement, retroperitoneal fibrosis, renal impairment, testis infiltration, breast involvement and central nervous system manifestations.^6^ A tissue biopsy is mandatory for histological confirmation and molecular profiling is required for therapeutic purposes. It is a clonal disorder associated with mitogen-activating protein kinase (MAPK) and BRAF V600E mutations.^7^

## Case Report

**Figure 1:**
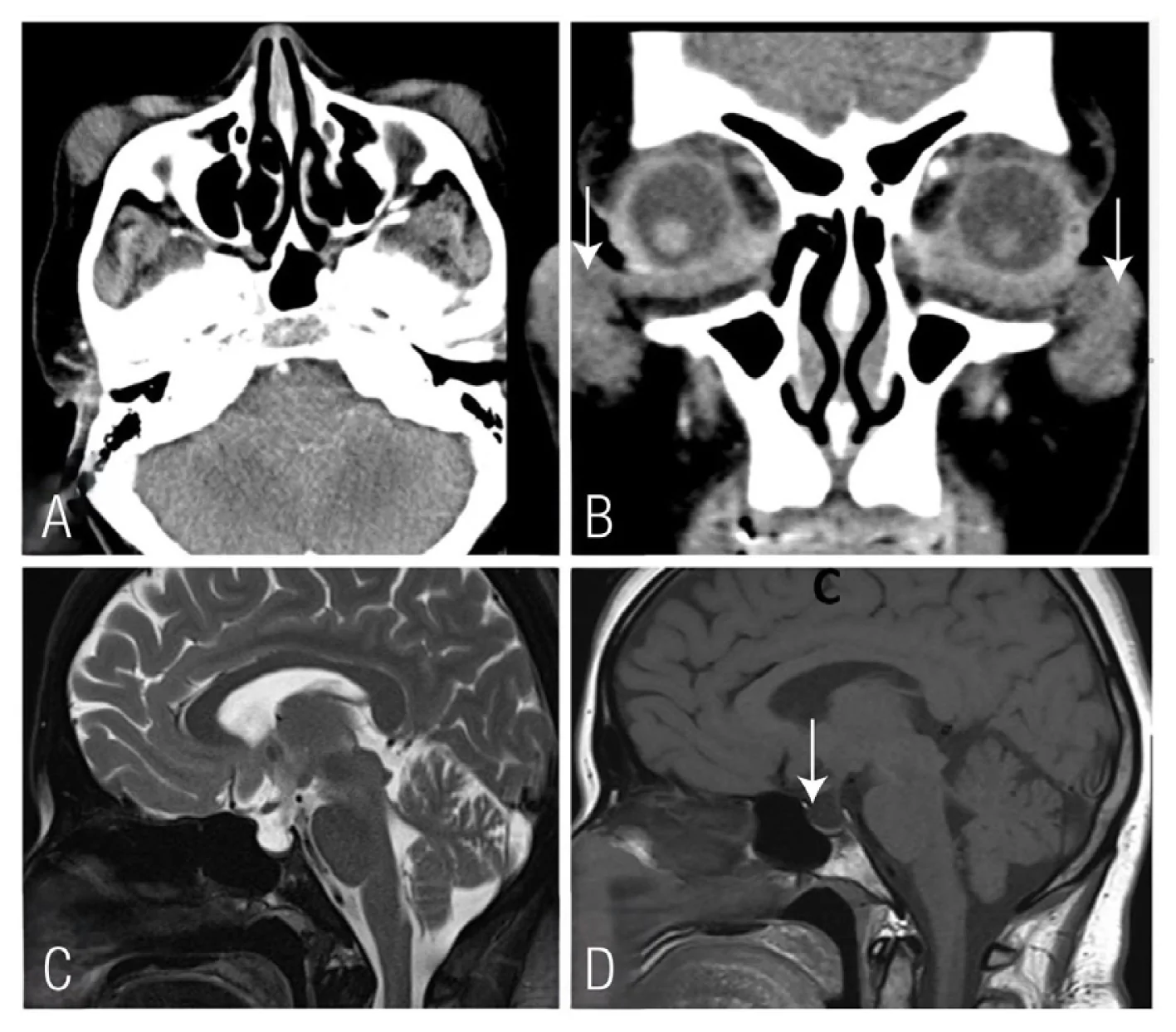
Computed tomography of the (**A**) axial and (**B**) coronal reformation showing soft tissue density mass lesions in prezygomatic areas bilaterally with no calcification or underlying bony involvement. Magnetic resonance imaging of the brain in sagittal (**C**) T2 and (**D**) T1 revealing non-visualisation of the pituitary gland with pituitary compressed along the sellar floor with pituitary fossa filled with cerebrospinal fluid signals and pituitary stalk reaching up to the sellar floor. Features are of empty sella syndrome.

A 49-year-old female presented to a tertiary care centre in Muscat, Oman, in 2012 with bilateral facial swellings. The right-side lesion was removed and histopathology showed a fibrohistiocytic lesion with foam cells. This was diagnosed as a benign fibrohistiocytic lesion. Later, she presented to the outpatient department with multiple other problems including fertility issues. She is known to have hypothalamic hypogonadism, diabetes insipidus, Hashimoto’s hypothyroidism, xanthelasmas and was recently diagnosed to have systemic lupus erythematosus (SLE). The facial lesion on the right side began to reappear and was again the size that was previously removed. A computed tomography scan of the head and neck showed bilateral soft tissue density masses in pre-zygomatic areas with no calcification or underlying bony erosions [Figure 1]. The plain radiography of the left lower limb showed sharply circumscribed sclerotic areas surrounding small lytic foci bilaterally in the distal femur and proximal tibia.

**Figure 2:**
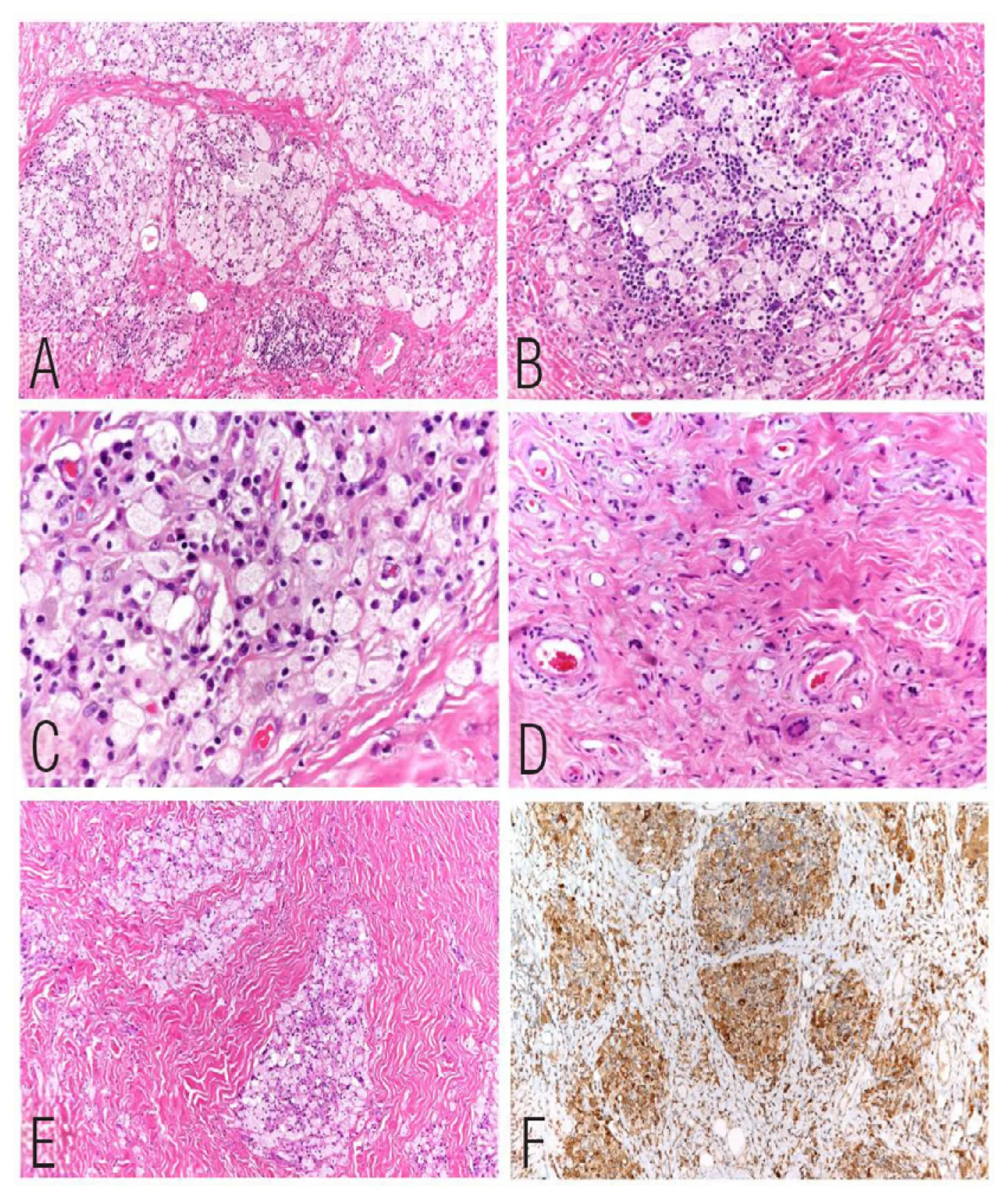
Haematoxylin and eosin stained section showing (**A**) foamy macrophages set in a spindle cell stroma and bundles of fibrous tissue in between spindle cells at ×4 magnification; (**B**) sheets of foamy macrophages with a sprinkling of lymphocytes at ×10 magnification; (**C**) sheets of foamy macrophages and the cells show abundant vacuolated cytoplasm with a central round nucleus at ×40 magnification; (**D**) spindle cells in the stroma with interlacing bundles of collagen and occasional Touton type of giant cells are present at ×40 magnification; and (**E**) low power view of macrophages and stroma at ×2 magnification. Immunohistochemical staining for CD68 antibody showing (**F**) positive immunoreactivity in the foamy cells and stromal cells at ×4 magnification.

A second surgery was done in March 2022 and the bilateral pre-zygomatic masses were removed. Histopathological examination showed features similar to that seen in the previous excision but with low cellularity and increased fibrosis. The histological examination showed groups, clusters and sheets of foamy macrophages set in a dense fibrous tissue with interspersed spindle cells and lymphoid aggregates with scattered giant cells with eosinophilic cytoplasm surrounded by multiple nuclei and clearing of cytoplasm at the periphery (Touton-type giant cells). These are also called xanthelasmatic giant cells due to their association with xanthelasmas. The histiocytic cells were positive for CD68 and negative for CD1a and S100 immunostains. The overall microscopic features were similar to a fibrohistiocytic lesion with an exuberant proliferation of foamy macrophages [Figure 2].

Patient consent was obtained for publication purposes.

## Discussion

ECD is a very rare chronic multisystem histiocytic neoplasm. The diagnosis is established by clinical, radiological and histological findings. Histiocytic disorders can be subdivided into Langerhans cell histiocytosis, non-Langerhans histiocytosis and malignant histiocytic disorders.^8^ Non-Langerhans histiocytoses are derived from the monocyte-macrophage lineage which are positive for CD68 and negative for CD1a. S100 staining is variable. ECD is a non-Langerhans histiocytic disorder characterised by multifocal osteosclerotic lesions of the long bones in addition to organ infiltration.^9^^,^^10^

There are few reported ECD related neoplasms in the retro-orbital area or facial bones, however, this case presented with bilateral facial soft tissue lesions. The reported endocrine abnormalities include hypopituitarism, hypogonadism and hypothyroidism. Hypogonadism is mainly confirmed by fertility issues, and this was seen later in this case who presented with hypogonadism, hypothyroidism and diabetes insipidus.^11^^,^^12^

Skin and soft tissue involvement is mainly in the form of xanthomas and xanthelasmas mostly in the head and neck area, predominantly the periorbital area, which is reported in approximately one-third of patients in multiple series. This was also seen in this case who had bilateral periorbital xanthelasmas.^12^

As this disease shares some common stem cells with haematopoietic neoplasms, approximately 10% of these tumours are associated with myeloid malignancies, for example, myeloid leukaemia. This must be taken into consideration in this case.^13^

The main reason for organ damage is fibrosis which results from fibroblastic proliferation in response to lymphokines and cytokines, which induce organ fibrosis and are not caused by the infiltration of histiocytes into the organs.^14^

Secondary involvement by autoimmune disease is common in ECD; approximately 40% of the cases have SLE-involvement and can have the typical serology for SLE. This is also typical in this case who presented later with features and serology of SLE.^15^

Treatment of ECD has drastically evolved since the better understanding of molecular aspects of the disease. Targeted therapies such as BRAF inhibitors and MEK inhibitors (drugs that block the MAPK pathway) have promising results yet considerable risks and side effects.^16^^,^^17^

## Conclusion

ECD remains a diagnosis of exclusion and should be considered when dealing with patients having multiple bone and soft tissue lesions with suggestive histology and multisystem involvement. Molecular studies hold a key position to make the diagnosis and provide hope for targeted therapy.
